# MicroRNA-142 is mutated in about 20% of diffuse large B-cell lymphoma

**DOI:** 10.1002/cam4.29

**Published:** 2012-09-18

**Authors:** Wiyada Kwanhian, Dido Lenze, Julia Alles, Natalie Motsch, Stephanie Barth, Celina Döll, Jochen Imig, Michael Hummel, Marianne Tinguely, Pankaj Trivedi, Viraphong Lulitanond, Gunter Meister, Christoph Renner, Friedrich A Grässer

**Affiliations:** 1 Institute of Virology, Saarland University Medical School66421, Homburg, Germany; 2 Institute of Pathology, Charité-Universitätsmedizin Berlin, Campus Benjamin Franklin12200, Berlin, Germany; 3 Institute of Pharmaceutical Sciences, ETH Zürich8093, Zürich, Switzerland; 4 Institute of Surgical Pathology, University Hospital Zürich8093, Zürich, Switzerland; 5 Department of Experimental Medicine and Pathology, Istituto Pasteur-Fondazione Cenci-Bolognetti, La Sapienza UniversityRome, Italy; 6 Department of Microbiology, Faculty of Medicine, Khon Kaen University40002, Khon Kaen, Thailand; 7 Biochemistry Center Regensburg (BZR), University of Regensburg93053, Regensburg, Germany; 8 Division of Oncology, Department of Internal Medicine-Oncology, University Hospital ZurichZurich, Switzerland

**Keywords:** Carcinogenesis, cellular biology, genomics, molecular genetics

## Abstract

MicroRNAs (miRNAs) are short 18–23 nucleotide long noncoding RNAs that posttranscriptionally regulate gene expression by binding to mRNA. Our previous miRNA profiling of diffuse large B-cell lymphoma (DLBCL) revealed a mutation in the seed sequence of miR-142-3p. Further analysis now showed that miR-142 was mutated in 11 (19.64%) of the 56 DLBCL cases. Of these, one case had a mutation in both alleles, with the remainder being heterozygous. Four mutations were found in the mature miR-142-5p, four in the mature miR-142-3p, and three mutations affected the miR-142 precursor. Two mutations in the seed sequence redirected miR-142-3p to the mRNA of the transcriptional repressor ZEB2 and one of them also targeted the ZEB1 mRNA. However, the other mutations in the mature miR-142-3p did not influence either the ZEB1 or ZEB2 3′ untranslated region (3′ UTR). On the other hand, the mutations affecting the seed sequence of miR-142-3p resulted in a loss of responsiveness in the 3′ UTR of the known miR-142-3p targets RAC1 and ADCY9. In contrast to the mouse p300 gene, the human p300 gene was not found to be a target for miR-142-5p. In one case with a mutation of the precursor, we observed aberrant processing of the miR-142-5p. Our data suggest that the mutations in miR-142 probably lead to a loss rather than a gain of function. This is the first report describing mutations of a miRNA gene in a large percentage of a distinct lymphoma subtype.

## Introduction

Diffuse large B-cell lymphoma (DLBCL) is the most frequently occurring form of non-Hodgkin's lymphoma (NHL) and accounts for an estimated 30% of all NHL cases worldwide [1, 2]. Moreover, DLBCL represents nearly 90% of aggressive B-cell lymphomas in the Western world. It is a heterogeneous disease, which consists of several histological subtypes, and if left untreated, it has an aggressive and fatal clinical course [1]. Today, the combined use of monoclonal anti-CD20 antibodies (rituximab) with CHOP-like poly-chemotherapy regimens has significantly altered the therapeutic landscape and improved clinical outcome [3]. Nevertheless, elderly patients, young patients with cofounding risk factors, or patients with early relapse of disease still face significant challenges and have an unacceptable low cure rate. Therefore, a better understanding of the molecular pathways involved in DLBCL pathogenesis and disease progression is needed to hopefully develop better tailored therapeutic approaches.

According to the most recent World Health Organization (WHO) lymphoma classification, DLBCL divides into subtypes based on genetic, immunological, morphological, and clinical features [1, 2]. Three molecular DLBCL subtypes can be identified by gene expression profiling (GEP) that correlates with clinical outcome [4]: germinal center B cell, like (GCB) DLBCL; activated B cell, like (ABC) DLBCL; and primary mediastinal B-cell lymphoma (PBML). The ABC-like cases have a poorer prognosis [5] when compared with GCB-like cases [6]. The latter occasionally features a translocation involving the *bcl-2* gene [4]. However, gene expression data do not capture all the biological parameters that influence diagnosis and response to therapy and are not yet included in clinical decision-making processes.

Therefore, new biomarkers with either predictive value or even therapeutic relevance are needed. MicroRNAs (miRNAs) might be suitable candidates as they are viewed as global regulators of almost all cellular pathways. They are composed of 18–23 nucleotides and bind mostly to target sequences within the 3′ UTR or in rare cases to the coding region of their target mRNAs thereby inhibiting protein expression. Some miRNAs are now recognized as oncogenes or tumor suppressors, and the miRNA profile can serve as a molecular signature of a particular tumor (for review, see [7, 8]). In addition, miRNA expression is associated with outcome in hematologic neoplasms and correlates with survival of DLBCL patients treated with rituximab-CHOP [9].

Deregulation of miRNA expression, for instance, via deletion or amplification of miRNA genes has been reported for a variety of tumors [10]. A germline mutation in the seed sequence of miR-125a at position +8 has been described [11] and was proposed to be a risk factor for breast carcinoma. This study involved a cohort of 72 cases of breast carcinoma from Belgium, and 282 Belgian and 587 Caucasian controls from the United States of America [12]. However, a recent study involving a total of 3145 breast cancer cases and 4114 controls showed no mutation at this miRNA [13]. Relatively little is known about somatic mutations directly affecting the mature miRNAs. In the complete sequence analysis of a single patient with acute myeloid leukemia, no mutations within the miRNAs were found [14]. A somatic point mutation in the precursor of human miR-33b not affecting the mature miRNA was observed in one of the 48 medulloblastoma cases, a highly aggressive brain tumor [15]. The sequence analysis of Colo-829, a cell line derived from a patient with a malignant melanoma, revealed a single point mutation in the central region of the stem loop structure of hsa-miR-518d [16]. Two cell lines and one primary pancreatic carcinoma specimen revealed two mutations each in the pri-miRNA regions of the two oncogenic miRNAs miR-21 and miR-155, again not affecting the mature miRNAs [17]. Permuth-Wey and colleagues recently published data suggesting that a single-nucleotide polymorphism in the precursor of miR-146a was associated with an increased risk for glioblastoma [18]. Robbiani et al. reported a reciprocal translocation in mature B-cell leukemia involving the miR-142 gene locus and the c-myc gene in transgenic, p53-deficient mice overexpressing AID [19]. A corresponding translocation in human aggressive B-cell leukemia involving the miR-142 locus (there called the bcl3 gene) and c-myc was described earlier [20]. In both cases, the translocation affected the levels of c-myc transcript, whereas point mutations in the miR-142 locus were not reported. In contrast to the scant information concerning mutations affecting mature miRNAs, polymorphisms in the binding sites for miRNAs have been reported to a larger extend (see, for instance, [21, 22]).

We had previously compared the miRNA profile of Epstein–Barr virus (EBV)-positive versus EBV-negative DLBCL by high-throughput sequencing of a miRNA cDNA library [23]. In one of the cases, we noted a mutation from T to C at position 8 in the seed sequence of miR-142-3p that was confirmed by DNA sequencing of the original tumor specimen. In that study, we found that various cellular miRNAs were up- or downregulated at least twofold due to EBV infection, but no significant change in the levels of miR-142-3p or -5p was observed [23]. MiR-142-3p is downregulated in acute myeloid leukemia [24] and inhibits migration and invasion of hepatocellular carcinoma lines [25]. Recently, the mRNAs coding for the small GTP-binding protein RAC1 and the adenylate cyclase 9 protein (ADCY9) were shown to be targets for miR-142-3p [25, 26]. RAC1 induction stimulates invasiveness of T-lymphoma cells [27] and is associated with poor prognosis of hepatocellular carcinoma [28]. ADCY9 activates RhoA, another GTPase with tumorigenic potential (reviewed in [29]). However, the function of ADCY9 in lymphomas is unclear. Recently, the mouse p300 gene was identified as a target for murine miR-142-5p [30].

The mutation in the seed sequence of miR-142-3p as identified by us generated three novel potential binding sites on the 3′ UTR of the mRNA coding for the transcriptional repressor ZEB2 (SIP1), which is, on one hand, involved in the switch of EBV latent to lytic replication [31], but also plays a role in cancer biology by regulating E-cadherin expression and the ensuing epithelial-to-mesenchymal transition (EMT) [32]. Here, we show that 11 of 56 DLBCLs suffered mutations in the gene-encoding miR-142 affecting either miR-142-5p, miR-142-3p, or the 87 nt precursor encompassing both miRNAs. Some of the miR-142-3p mutants acquire novel target regulatory functions vis-à-vis the ZEB1 or ZEB2 transcriptional repressors while some lose their ability to downregulate the known targets RAC1 and ADCY9. In contrast to the regulation of the mouse p300 gene/mRNA by miR-142-5p, we were not able to demonstrate a downregulation of human p300 by miR-142-5p, neither by testing the 3′ UTR nor by expression of miR-142 in human cells.

## Materials and Methods

### Patient samples

Tumor specimens of 40 DLBCL patients were obtained from the Institute of Pathology, Campus Benjamin Franklin, Charité-Universitätsmedizin Berlin, Germany (see Table S1). In addition, 16 DLBCL samples were obtained from the Institute of Surgical Pathology, University Hospital Zürich, 8093 Zürich, Switzerland. The diagnosis was confirmed by histopathology review according to the WHO classification criteria. Informed consent was obtained from all patients, and the use of the materials was approved by the local ethics committees.

### Immunohistochemistry

Sections from FFPE (formalin-fixed, paraffin-embedded) blocks were deparaffinized and antigenicity was retrieved by cooking in citrate buffer (100 mmol/L) for 5 min (BCL6 antibody) or 2 min (CD10 and IRF4 antibodies). Staining was performed using mouse monoclonal antibodies against BCL6 (1:50; Clone PG-B6p, Dako, Denmark), CD10 (1:25; Novocastra, Leica Microsystems, Germany), and IRF4 (1:25, clone Mum1p), kindly provided by B. Falini (Institute of Hematology, University of Perugia, Perugia, Italy). Binding was visualized with the APAAP Mouse REAL Detection System (Dako, Denmark) for the anti-CD10 antibody and REAL Detection System, Alkaline Phosphatase/RED (Dako, Denmark) for detection of anti-BCL6, and anti-IRF4 antibodies.

### Fluorescence in situ hybridization

Interphase fluorescence in situ hybridization (FISH) was performed on FFPE tissues using break-apart assays for MYC, BCL2, and BCL6 (all from Dako, Denmark), and LSI MYC/IGH and LSI BCL2/IGH dual-color dual-fusion translocation probes (all from Abbott/Vysis, USA). For signal detection an Axio Imager Z1 (Zeiss, Germany) together with Isis software (version 5.3.1, MetaSystems, Germany) was employed.

### Cell lines and plasmids

HEK 293T and U2932 (provided by P. Angel, identity confirmed by Schmitt and Pawlita [33], DKFZ, Heidelberg, Germany) were cultivated at 37°C, 5% CO_2_ in DMEM (Dulbecco's modified Eagle medium) or RPML (Roswell Park Memorial Institute) 1640 medium supplemented with 10% fetal bovine serum (Invitrogen, Carlsbad, CA) and 1× antibiotics (Sigma, St. Louis, MO) as described [24]. The vector pcDNA4hismaxCZEB2 for the expression of full-length ZEB2 [31] was a generous gift from Janet E. Mertz, University of Wisconsin School of Medicine and Public Health, Madison, WI, USA. The dual luciferase reporter plasmid pMIR-RL has been described elsewhere [23]. The 3′ UTR fragments of RAC1 (accession number: NM_018890), ZEB1 (accession number: NM_001128128), ZEB2 (accession number: NM_001171653), and ADCY9 (accession number: NM_001116) were polymerase chain reaction (PCR) amplified from human testis cDNA and ligated into the corresponding SacI and SpeI sites of pMIR-RL using the primers Rac1 SpeI 5′-GGG GTG TGT GTG ATC AAA GG-3′ and RAC1 SacI 5′-CCA CAA TTC TGC AAC TGT-C3′; ZEB1 SpeI 5′-GAT AAC TAG TTC TAG AAG GAA AAT AAA TTC TAA TTG AT-3′ and ZEB1 SacI 5′-GAT AGA GCT TAA GAC ATC AAA TAT TAG AAA TTA CTA-3′; ZEB2 SpeI 5′-GAT AAC TAG TGT ACA GTG TTA AGG CCT AAA AAC TGT G-3′ and ZEB2 SacI 5′-GAT AGA GCT CGT ACA GAA CTC ATT AAC TAC ATT CTT-3′; ADCY9 SpeI 5′-GAT AAC TAG TTC TAG AAG GAA AAT AAA TTC TAA TTG AT-3′ and ADCY9 SacI 5′-GAT AGA GCT CTA AGA CAT CTA AAT ATT AGA AAT TAC TA-3′. The human p300 3′ UTR (accession number: NM_001429) was amplified using the primers p300 SpeI 5′-GGA CTA GTG GCT GAG GCC TGT GAA GCC-3′ and p300 SacI 5′-CGA GCT CCC CCT TTT GTC CTC TGG-3′.

For the mutations of the binding sites of the miR-142-3p mutants in the 3′ UTR of ZEB2, the sites 1–3 as shown in Figure S1 were sequentially mutated as described [34] using the primer pairs ZEB2 mut1 5′-ACT CTA CTT ATG TAT AGA TCT AAA CTT TAA AAA AC-3′ and ZEB2 mut1rev 5′-GTT TTT TAA AGT TTA GAT CTA TAC ATA AGT AGA GT-3′; ZEB2 mut2 5′-TTT AAT TGC TCG AGA TCT AAT GCA TCA GTA TTA-3′ and ZEB2 mut2rev 5′-TAA TAC TGA TGC ATT AGA TCT CGA GCA CAT TAA A-3′; ZEB2 mut3 5′-CAT TTT AAA AAG GTG CCC GAG ATC TCA TAC ATC AG-3′ and ZEB2 mut3rev 5′-CTG ATG TAT GAG ATC TCG GGC ACC TTT TTA AAA G-3′. The binding sites for the mutant miR-142-3p-m1 and -m2 in the ZEB1 3′ UTR were mutated with the primers 5′ ZEB1-mut1-pmlI 5′-GTG AGA ACT TCT GCC ACG TGA AAT TCC CTT CA-3′ and 3′ ZEB1-mut1-pmlI 5′-TGA AGG GAA TTT CAC GTG GCA GAA GTT CTC AC-3′; 5′ ZEB1-mut2-Bgl 5′-TGT GAT TCC TGT TAG ATC TTG TGT AAA GTA-3′ and 3′ ZEB1-mut2-Bgl 5′-TAC TTT ACA CAA GAT CTA ACA GGA ATC ACA-3′, respectively. The mutations were detected using novel restriction sites (underlined) introduced in the seed region for miRNA binding.

### Sequence analysis

Genomic DNA from the Swiss tumor samples were purified from FFPE sections by the QIAamp DNA Mini Kit (Qiagen, Hilden, Germany) according to the manufacturer's protocol. In case of the German tumor samples, genomic DNA was extracted from fresh frozen tumor specimens, tonsils, and normal healthy peripheral blood mononuclear cells by standard protocols; miRNAs were purified using the miRVana miRNA Kit (Ambion, Austin, TX) according to the manufacturer's protocols. For the amplification of the miR-142 gene with the proof-reading Phusion DNA polymerase (New England Biolabs, Frankfurt/M., Germany), the primers 5′ miR142 EcoRI 5′-GGA ATT CGG GAT CTT AGG AAG CCA CA-3′ and 3′ miR142 Bam HI 5′-CGG GAT CCA TGG AGG CCT TTC AGG CAT-3′ were used. A typical 50 μL reaction consisted of 50–100 ng tumor DNA, 50 pmol each of the 5′ miR142 EcoRI and 3′ miR142 Bam HI primers, and 1 U of the Phusion polymerase. The following were the PCR settings: Step 1: 98°C, 10 min; Step 2: 98°C, 1 min; Step 3: 58°C, 1 min; Step 4: 72°C, 1 min; repeat Step 2 through 4, 35 times; Step 5: 72°C, 10 min. T/A-tailing was done using Taq polymerase (Sigma, Munich, Germany) in a 10 μL reaction using 5 μL of the above reaction, 2 μL dATP (10 mmol/L), and 5 U Taq polymerase. The resulting PCR products of 314 bp were T/A cloned into the vector pGEM-T Easy (Promega, San Luis Obispo, CA). For each sample, at least 10 clones were sequences using T7 and Sp6 primers. For those samples where mutations were found, additional samples were sequenced. For expression in eukaryotic cells, the EcoRI-BamHI-fragments were excided from the pGEM clones and inserted into the corresponding sites of the vector pSG5 (Agilent, Böblingen, Germany).

### Generation of ZEB2 antibodies

A rabbit antiserum against the C-terminus of ZEB2 was generated as described [34]. Shortly, a fragment encoding the 112 C-terminal amino acids of ZEB2 was PCR amplified from vector pcDNA4hismaxCZEB2 (see above) using the primers ZEB 5′ Eco (TAT GAA TTC ACC GAG CTG CTG ATG AAC CGG GC) and ZEB 3′ Sal (GCA GTC GAC TTA CAT GCC ATC TTC CAT ATT GTC TTC C) and cloned into the EcoRI/SalI sites of pGEX®-4T-1 (Promega, Mannheim, Germany). The corresponding GST-fusion protein was purified from *Escherichia coli* BL21/DE3 and used for the immunization of New Zealand white rabbits. For Western blotting, the serum was used at a dilution of 1:50 and visualized by the enhanced chemoluminescence (ECL) method as described [24]. For the detection of ZEB1, a polyclonal rabbit serum (H-106, sc-25388) was obtained from Santa Cruz, Heidelberg, Germany. A rabbit antibody against p300 (N-15) was purchased from Santa Cruz Biotechnology, Heidelberg, Germany (order #sc-584).

### Transfection and luciferase assays

Plasmid transfections for luciferase assays in 293T cells were performed with 0.2 μg of reporter construct and 0.8 μg miRNA expression plasmid in a 24-well plate using Nanofectin transfection reagent (PAA, Coelbe, Germany) as described by the manufacturer. Luciferase activity was measured 48 h post transfection using the Dual Luciferase Reporter Assay System as described by the manufacturer (Promega).

### Northern blot

Total RNA was extracted from cells using TriZol Reagent (Invitrogen, Carlsbad, CA) following the vendor's recommendations. Total RNA measuring 25 μg was routinely electrophoresed through 12% urea-polyacrylamide gel and then electroblot transferred to nylon membrane Hybond XL (Amersham) for 1 h at 2 mA/cm^2^. The membrane was EDC (1-ethyl-3-[3-dimethylaminopropyl]carbodiimid) chemically cross-linked as described [24]. Blots were hybridized with radioactive labeled antisense probe overnight and then washed twice for 15 min with 5× SSC (saline-sodium citrate), 1% SDS (sodium dodecyl sulfate) and twice for 15 min with 1× SSC, 1% SDS. As radioactive probes, we used appropriate RNA probes labeled with miRVana Probe construction kit (Ambion) according to the manufacturer's instructions.

### MicroRNA transfection assay and Western blot

MicroRNA expression using RNA mimics was done by transfecting 0.75 nmol per 10^6^ cells miR-142-3p-wt, miR-142-3p-m1, or miR-142-3p-m2 (Applied Biosystems/Ambion, Darmstadt, Germany). U2932 cells were transfected with lipid-based siPORT™ NeoFX™ Transfection Agent (Ambion) according to manufactures recommendation. Anti-miR miRNA Inhibitors – Negative Control #1 (Ambion) served as mock transfectant. After transfection cells were cultivated for 2 days, counted and protein extracts were generated by adding sample buffer (Promega). Protein levels were normalized according to the counted cell number. Western blot was performed as previously [24]. The relative levels of protein were determined by densitometric analysis of the signals for the tested proteins in comparison with the amount of β-actin stained on the same membrane with a commercially anti-human β-actin antibody (Sigma, Munich, Germany). The relative level of protein was determined from the scanned blots using the “Quantity One” software (BioRad Life Sciences, Heidelberg, Germany).

## Results

### Mutations affecting miR-142 are found in 20% of DLBCL analyzed

**Figure 1:**
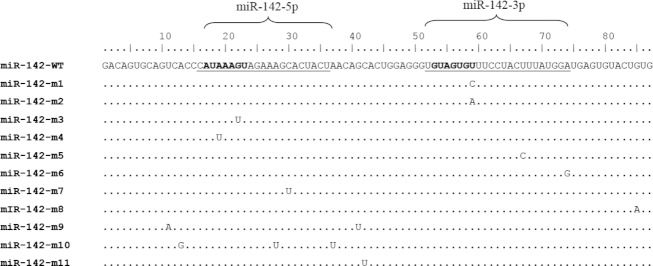
Mutations of miR-142 found in DLBCL. The sequence of the wild-type (WT)miR-142 precursor is shown on top, sequences of the mature miR-142-5p and -3p are underlined with the seed sequences shown in boldface. The mutations found in individual patients are shown below the wild-type sequence. Note that mutants m10 and m11 were found in one lymphoma.

In a previous study, we found no change in the expression levels of both miR-142-5p and -3p when we compared EBV-positive versus EBV-negative cases of DLBCL where both miR-142-5p and -3p were expressed at about equal ratios [24]. However, we detected a mutation affecting the seed region of miR-142-3p, which generated a potential novel binding site in the 3′ UTR of the ZEB1 mRNA and three potential novel binding sites in the 3′ UTR of the ZEB2 mRNA. Because ZEB2 plays a role in tumorigenesis [32], we wanted to see whether miR-142 was mutated in a larger number of DLBCL cases. We thus analyzed a total of 56 cases of DLBCL for the presence of mutations affecting miR-142 and also assayed the regulation of the above-mentioned 3′ UTRs vis-à-vis the mutant miRNA. We obtained PCR products from 56 cases that were subsequently analyzed. In the controls (DNA of 20 healthy blood donors and of 10 benign tonsils), no mutation affecting miR-142 was detected (data not shown). In addition, the sequences of the miRNAs obtained from the miRNA profiling data of 10 cases each of prostate carcinoma and normal prostate tissue that were previously obtained [35] also showed no mutation in the mature miR-142-3p or -5p (J. Szczyrba, pers. comm.). However, as depicted in Figure 1, we found single mutations within the 87 nt long precursor sequence for miR-142 in 11 (19.64%) of 56 cases of DLBCL. Of those, four single point mutations were present in the mature miR-142-5p or -3p. Two point mutations each affected the seed sequences of miR-142-5p (mutants designated -m3 and -m4 in Fig. 1) or -3p (mutants designated -m1 and -m2 in Fig. 1). The mutation designated m2 was detected in two samples. The mutations in m5 and m6 affected the mature miR-142-3p, and mutations in -m7 and -m10 were present in the mature miR-142-5p, but outside the seed sequence. Three additional mutations were found in the precursor outside the mature miRNAs. Of those, the mutant designated m9 had two base changes flanking miR-142-5p, and m10 had three mutations (two flanking and one mutation in the mature miR-142-5p outside the seed sequence). The mutations m10 and m11 occurred in one lymphoma. The mutations were at different sites within the miR-142 precursor; apparently, this patient suffered two individual mutations. In this sample, some wt-sequences were also detected which were probably derived from nontransformed stromal cells. We had access to normal tissue in four cases that featured a mutation in miR-142 (patients with mutants m3, m7, m9, and m10/11). Sequence analysis of the normal tissues showed no mutations indicating that the mutations in the tumors were probably somatically acquired. However, a larger number of patient's samples with mutations will need to be analyzed in the future.

We then asked whether the presence of the mutations correlates with clinical parameters or the lymphoma subtype. Of the 11 lymphomas with miR-142 mutation, five were of ABC and six of GCB type as determined by immunohistochemistry (IHC) [36]. Thus, the occurrence of the miR-142 mutations cannot be correlated to a certain DLBCL subtype. The same holds true for the different miR-142 mutations found. There is no association with either GCB or non-GCB subtype of DLBCL. We found no correlation to any parameter like age at lymphoma onset or gender. In addition, we could not identify any link to clinical data such as response to treatment or survival. However, all lymphomas harboring mutations in miR-142 showed expression of IRF4/Mum1 including those of GCB subtype by IHC (Table S1). IRF4/Mum1 is expressed in a relatively large number of malignant lymphoma and reported to be associated with good prognosis for B-cell chronic lymphocytic leukemia (B-CLL) (reviewed in [37]). However, another report found that expression of IRF4 was associated with a poor prognosis for B-cell chronic lymphocytic leukemia (CLL)/small lymphocytic lymphoma (SLL) [38]. So far, there is no clear correlation between the expression of IRF4 and the prognosis of DLBCL [39]. The percentage of mutations found in the sequence reactions of the individual lymphomas is shown in Table S2.

### Point mutations in the seed sequence of miR-142-3p generate novel target sites in the ZEB1 and ZEB2 3′ UTRs

**Figure 2:**
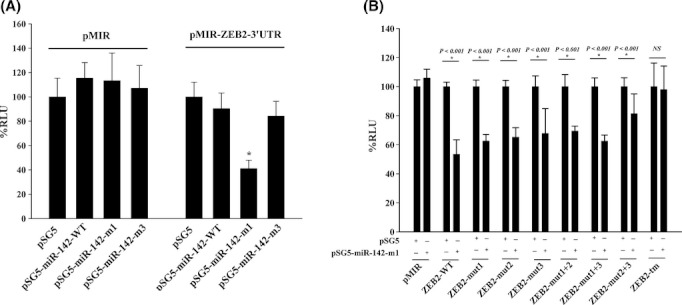
Downregulation of the ZEB2 3′ UTR by miR-142-3p mutant m1. (A) An empty luciferase reporter (“pMIR”) or a reporter containing the ZEB2 3′ UTR (“pMIR-ZEB2-3′ UTR”) and expression vectors for miR-142-3p and the mutants -m1 and -m3 were coexpressed in HEK 293T cells as indicated. (B) Luciferase reporter vectors containing mutated binding sites in the ZEB2 3′ UTR were tested for responsiveness to miR-142-m1. Columns represent the mean values of three independent experiments carried out in duplicate (± SEM).

A bioinformatical analysis using the “Custom TargetScan” algorithm (http://www.targetscan.org/) employing the mutated seed sequence of miR-142-3p-m1 shown in Figure 1 indicated that the 3′ UTR of the ZEB2 mRNA contained three potential novel binding sites for m1. This is shown in Figure S1. We therefore obtained the complete 3′ UTR of ZEB2 by PCR amplification and subsequently generated a luciferase reporter construct as previously described [24]. The luciferase reporter analysis shown in Figure 2A, right panel, demonstrated that m1 but not the miR-142-3p wild-type nor the miR-142-3p-m3 mutant had a significant effect on the reporter. We observed a significant downregulation of the reporter construct by m1 by about 52% (*P* = 3.5E-08). None of the miR-142 constructs had an effect on the empty pMIR reporter vector (Fig. 2A, left panel). Next, we analyzed which of the potential binding sites on the ZEB2 3′ UTR were used by m1. For this purpose, either single mutations or the possible combinations of the potential binding sites were generated. Mutations of the single sites had no effect (Fig. 2B, left panel), and only the mutation of all three potential binding sites resulted in a complete loss of reactivity indicating that all three sites were used (Fig. 2B, right panel). The sequential introduction of the mutations led to an increased loss of reactivity as compared with the single mutants or the wt-3′ UTR indicating that there was a cooperative effect of the binding sites.

**Figure 3:**
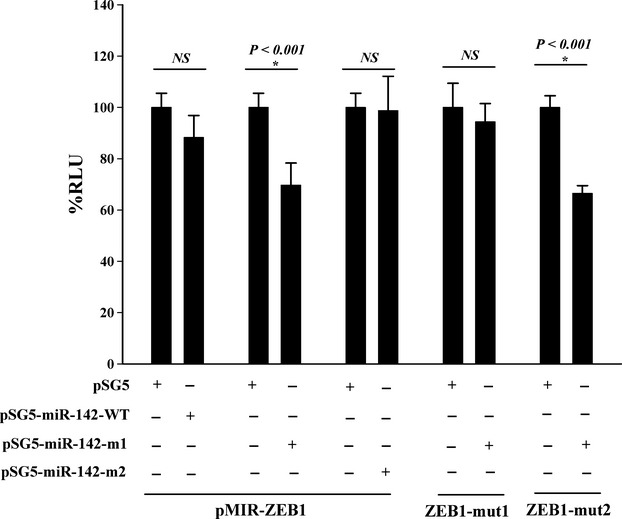
Downregulation of the ZEB1 3′ UTR by miR-142-3p-m1 but not m2. (A) The empty luciferase reporter construct (pMIR, left panel) or a reporter containing the wild-type ZEB1 3′ UTR (right panel) was coexpressed with vectors for miR-142-3p and the mutants -m1, -m2, and empty pSG5 vector as indicated. (B) Reporter vectors with mutated binding sites in the ZEB1 3′ UTR were coexpressed in HEK 293T cells with expression vectors for miR-142-wt, -m1, and -m2 in the indicated combinations. Coexpression of pSG5 served as control. Mean values were derived from three independent experiments carried out in duplicate (± SEM).

We then assayed the effect of m1 and m2 on the ZEB1 3′ UTR and found that only m1 downregulated this reporter. The mutation of the two potential binding sites for m1 on the ZEB1 3′ UTR revealed that binding site 1 was used by the mutants (Fig. 3). As about 15% of all DLBCLs are EBV positive, we asked whether any of the EBV-encoded miRNAs (for review, see [40]) could potentially target the ZEB1 or ZEB2 3′ UTR. The “TargetScan” algorithm predicted binding sites for both EBV-miR-BART7 and -BART22 on the ZEB2 but not on the ZEB1 3′ UTR. However, neither of the two EBV-miRNAs had any effect on the empty vector or the pMIR-ZEB2-3′ UTR construct (Fig. S2).

### Seed-sequence mutations of miR-142-3p result in downregulation of ZEB2 protein expression

**Figure 4:**
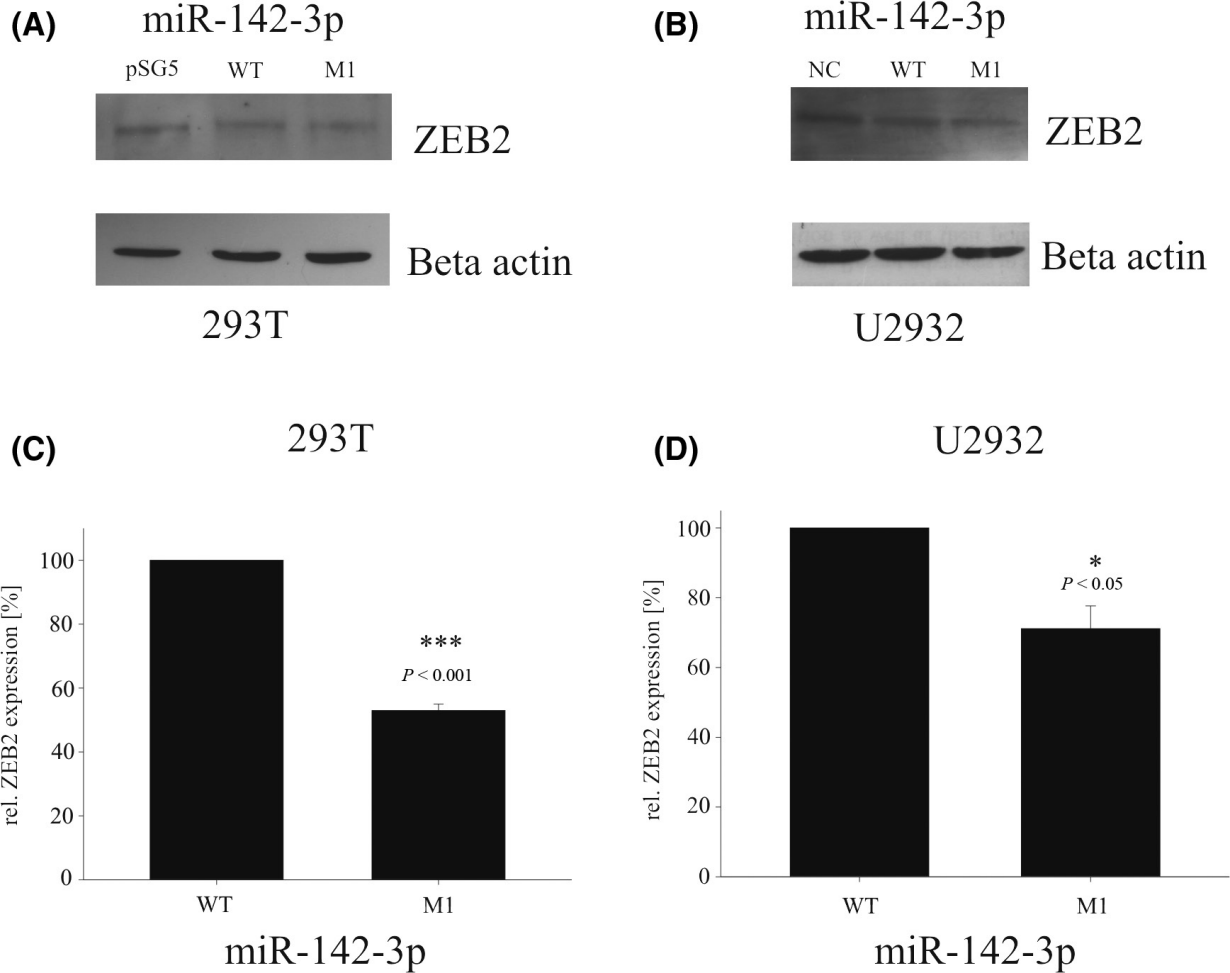
Downregulation of the ZEB2 protein by miR-142-3p mutant m1. HEK 293T cells (A) were transfected with empty pSG5 vector, pSG5-miR-142-3p-wt, or the m1 mutant as indicated. The levels of β-actin stained on the same blot were used as input control. The graph shown in (C) depicts the quantification of three individual assays. The DLBCL cell line U2932 was transfected with RNA mimics using the miR-142-3p-wt or a scrambled RNA mimic (“NC”) as controls as indicated (C). The graph in (D) depicts the quantification of the downregulation of ZEB2 in four individual experiments. ZEB2 was visualized by Western blotting using a polyclonal rabbit serum directed against the C-terminus of ZEB2.

To show that the mutation in the miR-142-3p-m1 indeed affect the expression level of their mRNA targets, polyclonal rabbit antibodies against the ZEB2 protein were generated using a bacterially expressed GST-ZEB2 fusion protein as previously described [41]. The immune serum was tested in cell extracts of 293T cells expressing a haemagglutinine (HA)-tagged ZEB2 expression vector [31] as control. Expression of the recombinant ZEB2 could both be verified using a HA-specific antibody as well as the ZEB2-specific rabbit serum (data not shown). We then tested the expression level of ZEB2 in 293T cells transfected with empty vector, the pSG5-miR-142-wt vector, and pSG5-miR-142-3p-m1. As shown in Figures 4A and S5, the vector expressing miR-142-3p-wt did not change the levels of ZEB2 in 293T cells, whereas the expression of the mutant m1 clearly led to a reduction of about 40% in the amount of endogenous ZEB2. We then analyzed the EBV-negative U2932-DLBCL cell line for the influence of miR-142-3p-wt or the -m1 mutant. Here, we used a RNA mimic either for the wild-type or the m1 mutant because the transfection efficiency using pSG5 expression vectors was too low (data not shown). As shown in Figure 4B, neither the negative control (“NC”) nor the wild-type mimic changed the level of ZEB2 while we observed a reduction of about 30% using the mutant m1 mimic.

We then used a commercially available antiserum against ZEB1 (H-106, Santa Cruz) and found no expression of this protein in the U2932 cell line (data not shown). Therefore, the impact of the miR-142 mutants on ZEB1 expression was not pursued further.

### Mutations in the seed sequence of miR-142-3p result in a loss of responsiveness of the RAC1 and the ADCY9 3′ UTRs

**Figure 5:**
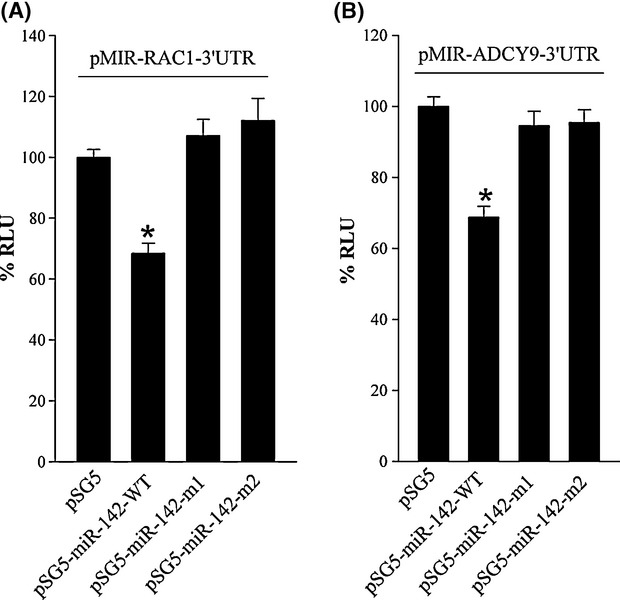
MiR-142-3p mutants m1 and m2 lose their potential to downregulate the RAC1 and ADCY9 3′ UTRs. (A) Luciferase constructs featuring the binding regions for miR-142-3p on the RAC1 3′ UTR (“pMIR-RAC1-3′ UTR”) (A) or the ADCY9 3′ UTR (B) were assayed with empty pSG5 and pSG5 expression vectors for miR-142-3p-m1 and -m2 after cotransfection into HEK 293T cells in the indicated combinations. The graphs represent eight independent assays carried out in duplicate (± SEM).

It was previously shown that the 3′ UTR of the mRNAs for the small GTP-binding protein RAC1 as well as the ADCY9 represents targets for miR-142-3p [18, 26]. We therefore asked whether the mutation in the seed sequence of miR-142-3p-m1 or -m2 had an influence on the downregulation of RAC1 by this miRNA. For this purpose, a luciferase reporter construct featuring the 3′ UTR of RAC1 was generated. When assayed with the various expression vectors, we found that miR-142-3p-wt downregulated the luciferase activity by about 35% (*P* = 2.4 exp 7), while both -m1 and -m2 had lost their ability to influence the RAC1 3′ UTR (*P* = 0.23 and *P* = 0.133, respectively) (Fig. 5A). Furthermore, we found that both mutants also lost their potential to downregulate the ADCY9 3′ UTR, while miR-142-3p-wt clearly had an effect toward this 3′ UTR (*P* = 0.000021 for miR-142-3p-wt, *P* = 0.027 for -m1, and *P* = 0.28 for -m2) (Fig. 5B).

### Mutations in miR-142 affect correct processing

**Figure 6:**
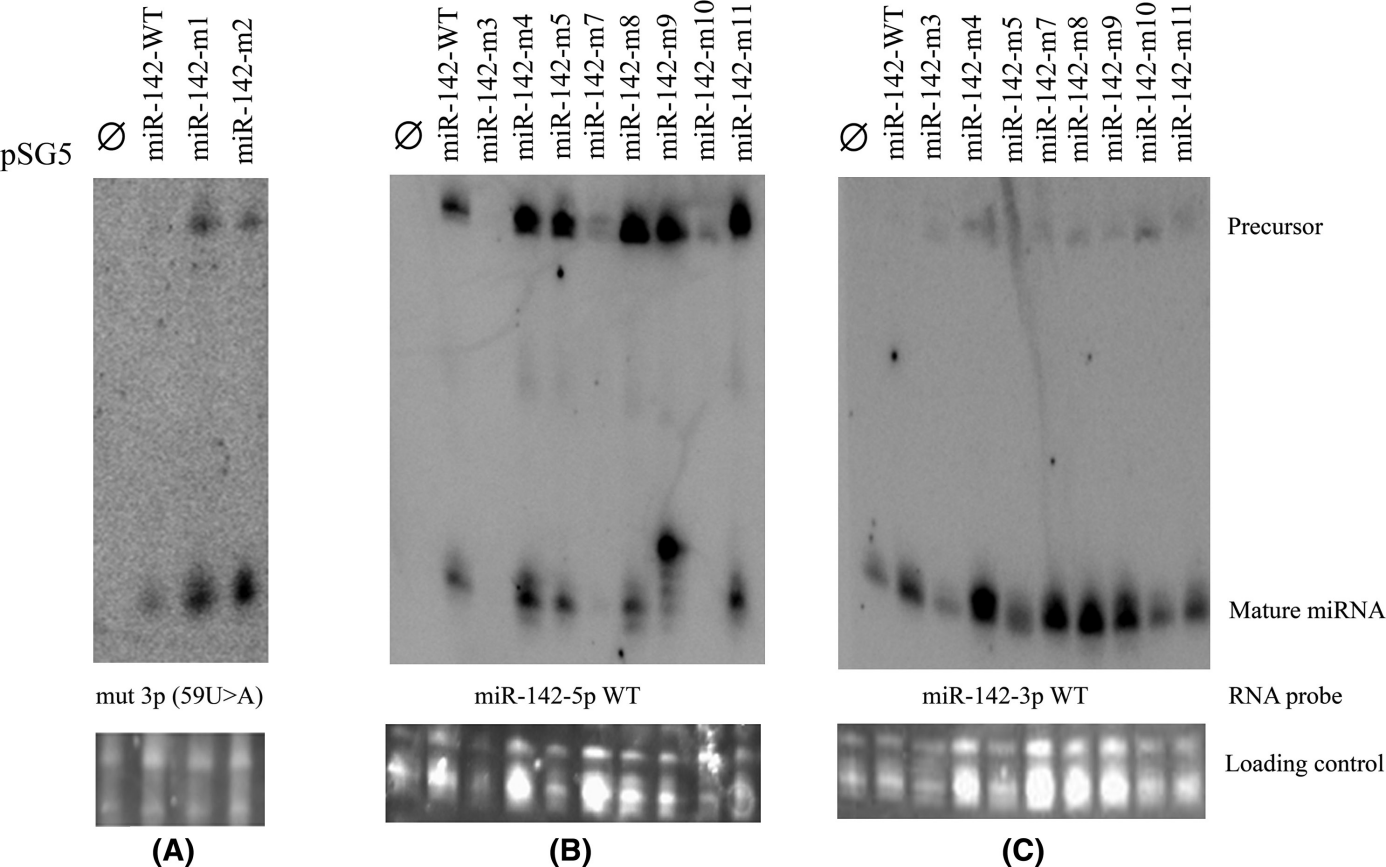
Expression of miR-142 mutants. The various miR-142-3p, -5p, or precursor mutants were expressed in HEK 293T cells. The expression of miR-142-3p m1 and m2 (A and C) or -5p (B) was assayed by Northern blotting using the indicated probes. The position of the precursor and the mature miRNAs are also shown. The bottom lane shows the band of the tRNA input control.

We then assayed whether the mutants that did not affect the mature miR-142-5p or -3p showed any difference in their processing. Mutants were transiently expressed using the pSG5 expression vector and analyzed by Northern blotting. For reasons unknown, we were not able to sub-clone the mutant m6 into the pSG5 expression vector despite repeated attempts. As shown in Figure 6, all mutants with the exception of m9 were properly processed to the mature miR-142-5p or -3p. We initially observed no or low signals for miR-142-m3-5p, m7-5p, and m10-5p. These were therefore reanalyzed with perfectly matching antisense probes. As shown in Figure S3, these 5p-mutants were also expressed. We noted a more complete conversion of the -3p precursors as compared with the -5p precursors. The mutant m9 showed correct processing to the mature miR-142-3p but exhibited a somewhat larger “mature” miR-142-5p. To ascertain that the miR-142-5p and the other mutants were processed correctly, we carried out a Northern blot analysis comparing the transiently expressed miR-142-5p-wt, -m1, -m3, and -m9 along with RNA from U2932 DLBCL cells [24]. As shown in Figure S4, miR-142-5p-wt and m1 migrated to the same position as the endogenous miR-142-5p from the U2932 cells. As already shown in Figure 6, m3 was not recognized using the probe for miR-142-5p-wt, and the m9 mutant again migrated to a higher position. The correct processing to miR-142-3p was previously demonstrated [42].

### Human p300 is not a target for miR-142-5p

**Figure 7:**
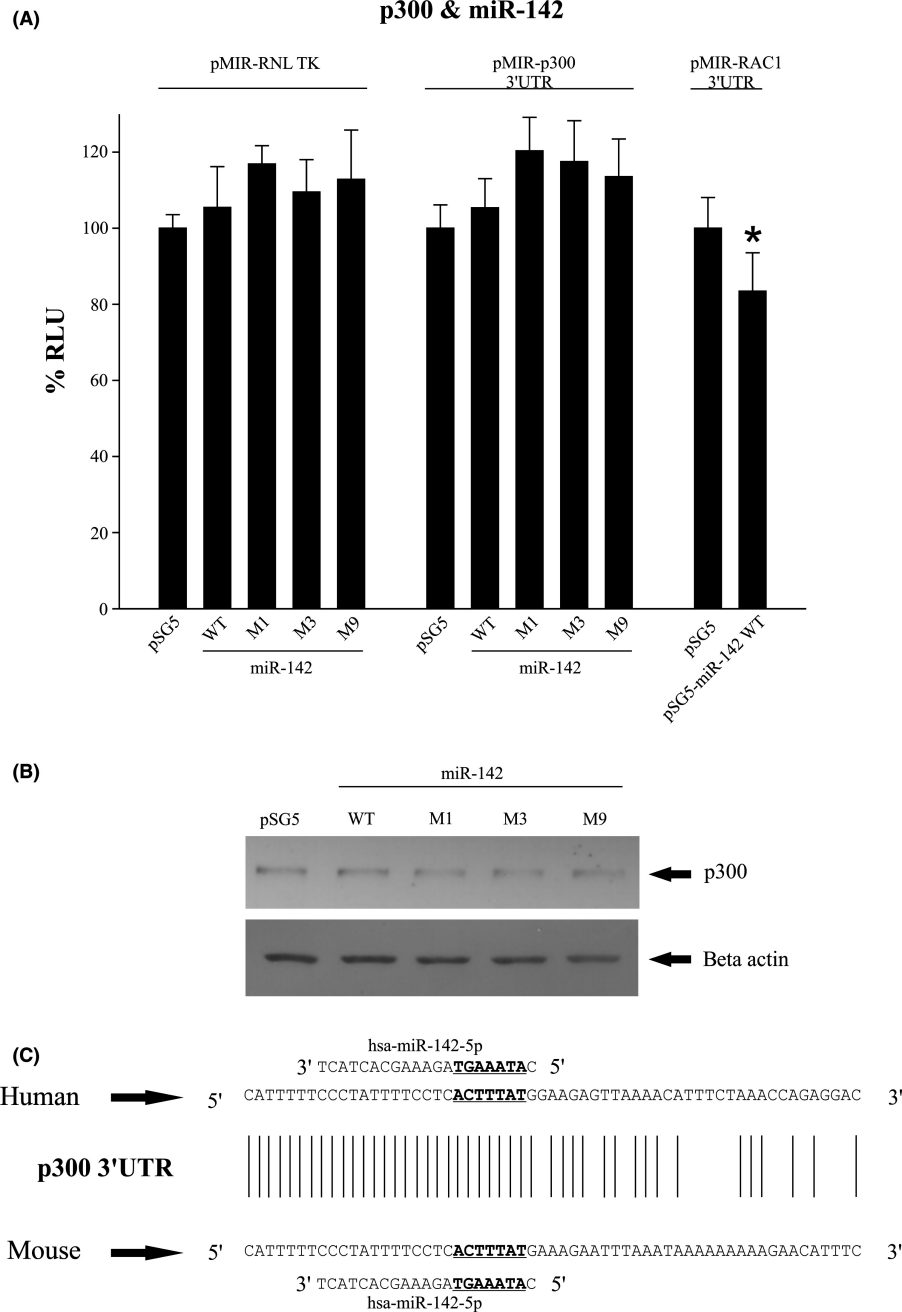
The human p300 mRNA is not a target for miR-142-5p. (A) Luciferase constructs featuring the binding regions for miR-142-3p on the p300 3′ UTR (“pMIR-p300-3′ UTR”) and the empty pMIR-RNL-TK vector were cotransfected with the indicated pSG5 miR-142 expression vectors. The pMIR-RAC1-3′ UTR reporter (see Figure 5) served as a positive control. The graphs represent four independent assays carried out in duplicate (± SEM). (B) HEK293T cells were transiently transfected with the indicated pSG5 miR-142 expression vectors and assayed 48 h after transfection in a Western blot. p300 was visualized by the ECL method using a rabbit antibody and an appropriate secondary antibody. The level of β-actin served as a loading control. (C) Comparison of the known mouse p300 and the putative human p300 binding site for miR-142-5p. The seed sequence is indicated by bold letters, the binding region for miR-142-5p by a bar.

Sharma et al. had previously shown that the mouse p300 gene is a target for miR-142-5p. We therefore tested the 3′ UTR of the human p300 gene (accession number NM_001429) for its responsiveness to miR-142-5p-wt and of the mutants m1 (mutation in the miR-142-3p), m3 (mutation in the miR-142-5p seed sequence), and the incorrectly processed 5p mutant m9. As shown in Figure 7A, the p300 3′ UTR was not responsive to either the miR-142-wt or any of the mutants, while the RAC1 3′ UTR assayed in parallel was clearly downregulated by miR-142-wt. The various plasmids were also transiently transfected in 293T cells and tested for their ability to downregulate the p300 protein. As shown in Figure 7B, the p300 protein was not affected by the expression of either miRNA as compared with the empty vector control. The mature human and the mouse miR-142-5p are identical. The alignment of the predicted and known binding sites for miR-142-5p in the human and the mouse p300 3′ UTRs, respectively, are shown in Figure 7C. As can be seen, the mouse sequence is divergent immediately 5′ to the seed sequence from the human gene which might explain this discrepancy.

## Discussion

To the best of our knowledge, this is the first report demonstrating that mutations affecting the mature sequence of a miRNA occur in a sizable proportion of a given lymphoma subtype. In particular, we observed both a gain of function and a loss of function caused by this miRNA mutation. We could clearly demonstrate the downregulation of the ZEB2 mRNA by the miR-142-3p mutant m1 (Fig. 1) both in the reporter assays using the ZEB2 3′ UTR (Fig. 2) and on the ZEB2 protein level (Fig. 3). We could also demonstrate an effect of the mutants miR-142-3p-m1 and -m2 on the 3′ UTR of the ZEB1 mRNA. As ZEB1 was not detectable with commercially available antibodies in the U2932 DLBCL cell line assayed, we did not further test the effect of the mutants on ZEB1 protein. However, as the mutations occurred both in the mature miR-142-5p and -3p form, we assume that the observed gain of function for miR-142-3p on the ZEB1 or ZEB2 3′ UTR was a fortuitous event. At this time, we do not know whether this “gain of function” is relevant for the induction of DLBCL. However, we wanted to show that the mutated miRNA was functional toward its novel target but unable to downregulate known targets like RAC1 or ADCY9 (see also below). In addition, there was no significant difference in the levels of ZEB1 or ZEB2 mRNA in the tumors analyzed (data not shown).

Conversely, we were able to demonstrate that the mutations in the seed sequence of miR-142-3p destroyed the ability of the miRNA to inhibit the RAC1 mRNA. RAC1 is a member of the Rho family of small GTPases and has been strongly implicated in tumorigenesis [43, 44]. It is involved in B-cell signaling through the CD40 receptor after activation of the RAS pathway [45] and was found to be activated in anaplastic large T-cell lymphomas [46]. RAC1 was shown to be a target of miR-142-3p in hepatocellular carcinoma cells [26]. A separate report demonstrated that overexpression of RAC1 in p53-deficient B- and T-lymphoma cells resulted in an increased lymphoma cell proliferation [18]. Another recent report showed that RAC1 and the proto-oncogene BCL-2 form a complex and that RAC1 supports the antiapoptotic activity of BCL-2 [47]. BCL-2 is sometimes activated by chromosomal translocations in DLBCL [48]. It is thus conceivable that at the seed-sequence mutations in miR-142-3p result in a release of RAC1 suppression with a subsequent block in apoptosis, for instance via binding to BCL-2. It is thus conceivable that some of the mutations described in this communication contribute to the induction of DLBCL via a loss of RAC1 inhibition by the mutated miR-142-3p.

The two mutants m1 and m2 which show a “gain of function” vis-à-vis the ZEB1 and ZEB2 3′ UTRs also lose their ability to downregulate a second known target of miR-142-3p, the ADCY9 3′ UTR. A reduced inhibition of ADCY9 by the mutated miR-142-3p would result in increased cAMP levels. For DLBCL, it was shown that increased levels of cAMP are proapoptotic in DLBCL. An upregulation of cAMP levels via a reduced inhibition of PKA targeting is thus counterintuitive to its possible role in DLBCL tumorigenesis. Zhnag et al. have shown that high levels of miR-375 and low levels of miR-142-5p are a predictor for the outcome of gastric carcinoma [49]. Furthermore, reduced levels of miR-29a and miR-142-3p are shown to promote growth of acute myeloid leukemia cells, while overexpression leads to a differentiation of myeloid cells with reduced growth properties [50]. At least in some cases of DLBCL, the mutations in miR-142-3p/5p might also influence the tumor cell growth via reduced differentiation.

The aim of the experiments was to show that both a gain of function and a loss of function can be observed for the mutants tested. We did not observe mutations in other miRNAs, which indicates that the mutations are specific for miR-142. We again hypothesize that the miR-142 mutations will most likely result in a loss rather than a gain of function. The nature of the genes that are regulated by miR-142 in DLBCL is unknown and needs to be elucidated. Lv et al. have shown that miR-142-3p plays an oncogenic role in T-lineage acute lymphoblastic leukemia (T-ALL) [51]. We have also found a downregulation of miR-142-3p in EBV-positive nasal NK/T-cell lymphoma [52]. On the other hand, miR-142 might have a tumor-suppressive role in B cells, and the loss of function could then contribute to the induction of DLBCL.

We also analyzed the expression levels of the cloned miR-142 genes and found no obvious change in the expression of either miR-142-3p or -5p in the various clones (Fig. 6). In the case of miR-142-5p-m9, we observed aberrant processing of the miRNA which led to the generation of a larger final product. Because there are no confirmed human miR-142-5p targets, it was not possible to test whether the larger product (or the other mutants affecting the mature miR-142-5p) was still able to regulate a potential target. In addition, it is unclear whether this aberrant miRNA still contained the complete sequence present in miR-142-5p or whether, for instance, the 5′ nucleotides essential for target recognition were absent. It is possible that the addition of nucleotides at the 5′ end also leads to a change in target recognition, most likely in the loss of recognition of (a) target(s). Finally, when we assayed some of the miR-142-5p mutants on the human p300 3′ UTR, we did not find a downregulation by either the wild-type or the mutant miRNAs. Likewise, the p300 protein was not reduced in transiently transfected cells. This is in contrast to recently published data which identified the 3′ UTR of the mouse p300 gene as a target for miR-142-5p [30].

Lohr et al. described the whole exome sequencing of protein-encoding genes in 55 cases of DLBCL [53]. They found a variety of mutated genes, for example, MYD88, CARD11, or EZH2 [53]. They show that while some genes like bcl2 were probably mutated due to the activity of the AID (activation-induced cytidine deaminase) gene, the others were probably caused by AID-independent mechanisms. As none of the C-residues in the miR-142 mutants described in the present paper confers to the WRCY (tryptophan-arginine-cysteine-tyrosine) motif used by AID, we assume that the mutations were not caused by AID but by another mechanism active in DLBCL. In that study, the presynaptic cytomatrix protein (PCLO, *piccolo*) gene had the highest number of cases with 17 (32%) of 55 patients showing 23 different mutated sites in the PCLO gene, while other genes had lower but still significant numbers of changes within the genes analyzed. The findings by Lohr et al. reinforce the notion that the same set of genes is mutated in DLBCL as previously described by Morin et al. [54] and Pasqualucci et al. [55]. Thus, miR-142 can be added to the growing number of genes that are mutated in DLBCL.

In our previous study on miRNA expression in DLBCL, we found that both miR-142-3p and -5p were expressed at comparable levels (1.1% and 0.8%, respectively, of the total miRNA count) and they may therefore be of equal importance for the regulation of cellular targets [24]. A comparison of the miR-142-5p levels in Burkitt's lymphoma versus DLBCL showed that primary Burkitt's lymphoma expresses significantly less miR-142-5p than primary DLBCL [56]. Since no targets are known for miR-142-5p, we can only speculate about the consequences of the mutations affecting miR-142-5p. It might be possible that both miR-142-3p and -5p are involved in the regulation of pathways that eventually converge at (a) final target(s). As described by Wu et al. [26], miR-142-3p appears to be a negative regulator of cell growth. In addition, the putative tumor suppressor nasopharyngeal carcinoma-associated gene 6 (NGX6), when expressed in colon carcinoma cell lines, induced the expression of miR-142-3p, whereas miR-146a, which is known to be induced via NFkb by the EBV oncogene LMP1 [57], was reduced by NGX6 [58]. Further experiments will be necessary to analyze the function of miR-142-3p and -5p for lymphoma cell growth. Likewise, it is important to identify targets for miR-142-5p. At present, we favor the idea that the mutations within either miR-142-3p or -5p result in a loss of function, which might result in a growth stimulation in DLBCL.

## Supporting Information

**Figure S1.** Schematic representation of the potential binding sites for miR-142-m1 and miR-142-m2 in the ZEB1 and ZEB2 3′ UTRs in the luciferase reporter constructs. The predicted binding sites, the seed sequences, and the mutated seed sequences are shown.

**Figure S2.** The EBV miRNAs ebv-miR-BART7 and ebv-miR-BART22 do not regulate the ZEB2 3′ UTR. The empty luciferase reporter construct (left panel) or the pMIR reporter containing the wild-type ZEB2 3′ UTR (right panel) was coexpressed with vectors for ebv-miR-BART7 or ebv-miR-BART22. Coexpression of pSG5 served as control. Mean values were derived from three independent experiments carried out in duplicate (± SEM).

**Figure S3.** Northern blot analysis of the miR-142-mutants -m3, -m4, -5, and m10. The indicated mutants were expressed in HEK 293T cells and assayed in a Northern blot using the indicated probes that perfectly matched the mutated miRNAs. The bottom lane shows the band of the tRNA input control.

**Figure S4.** Comparison of transiently transfected miR-142-5p mutants with endogenous miR-142-5p. HEK293T cells were transiently transfected with empty psg5 vector and the mutants m1, m3, m7, and m9 as indicated. RNA derived from the U2932 cell line was analyzed in parallel. As shown in Figure 6, the miR-142-5p-wt probe did not yield a signal for m3 or m7. The bottom lane shows the band of the tRNA input control.

**Figure S5.** Downregulation of the ZEB2 protein by miR-142-3p mutant m1. HEK 293T cells were transfected with empty pSG5 vector, pSG5-miR-142-3p-wt or the m1 mutant as indicated. Shown are the individual experiments used for the statistical analysis depicted in Figure 4. The levels of β-actin stained on the same blot were used as input control. The relative downregulation, shown below each blot, was determined by scanning of the blots and then compared to the β-actin input control using the “Quantity One!” program, BioRad, Heidelberg, Germany.

**Table S1.** Features of the tumours with mutations in miR-142 (OS: overall survival; BA: break-apart assay; cell of origin: determined according to Ref. 36).

**Table S2.** miR-142 mutations found in 56 DLBCLs.
