# Supplementary figures and images for: MicroRNA-142 is mutated in about 20% of diffuse large B-cell lymphoma
Source: Cancer Med. 2012 Sep 18;1(2):141–55. doi: 10.1002/cam4.29 (PMC3544448; doi:10.1002/cam4.29)

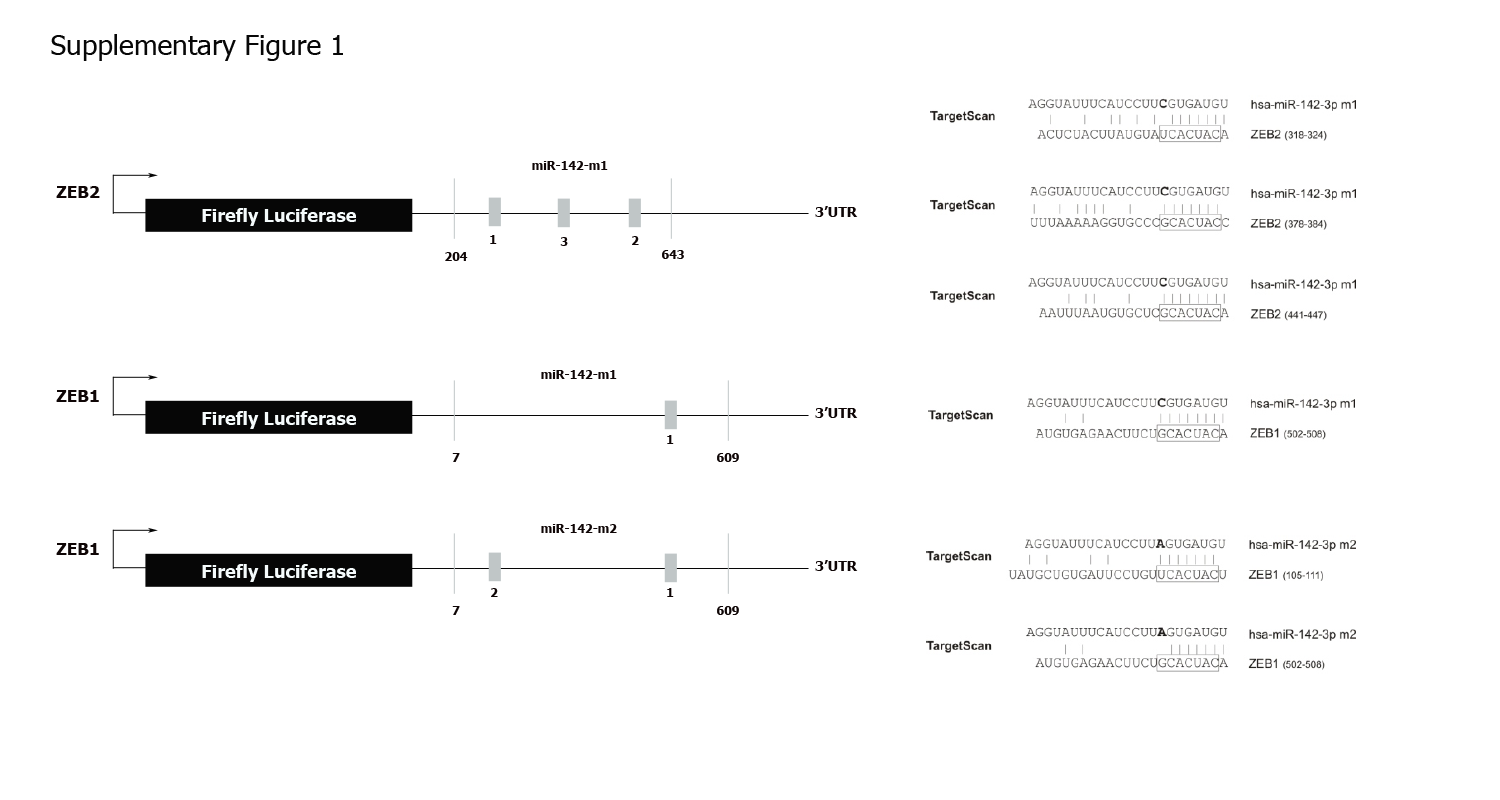

Supplement: Supplementary file 1 [file cam40001-0141-SD1.tif]

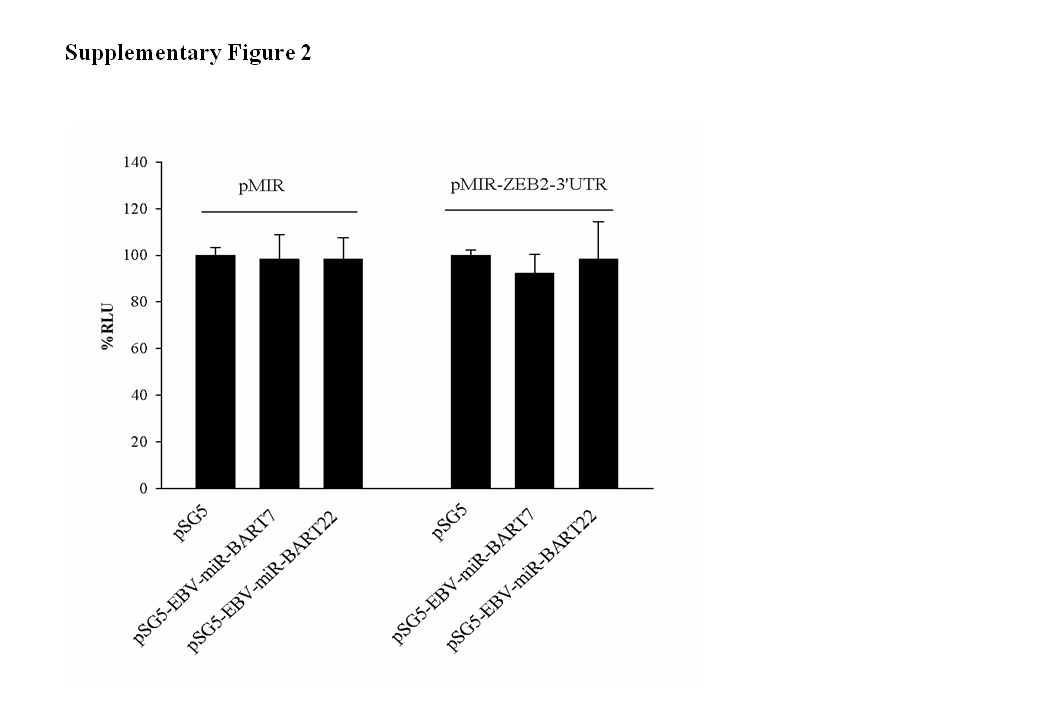

Supplement: Supplementary file 2 [file cam40001-0141-SD2.tif]

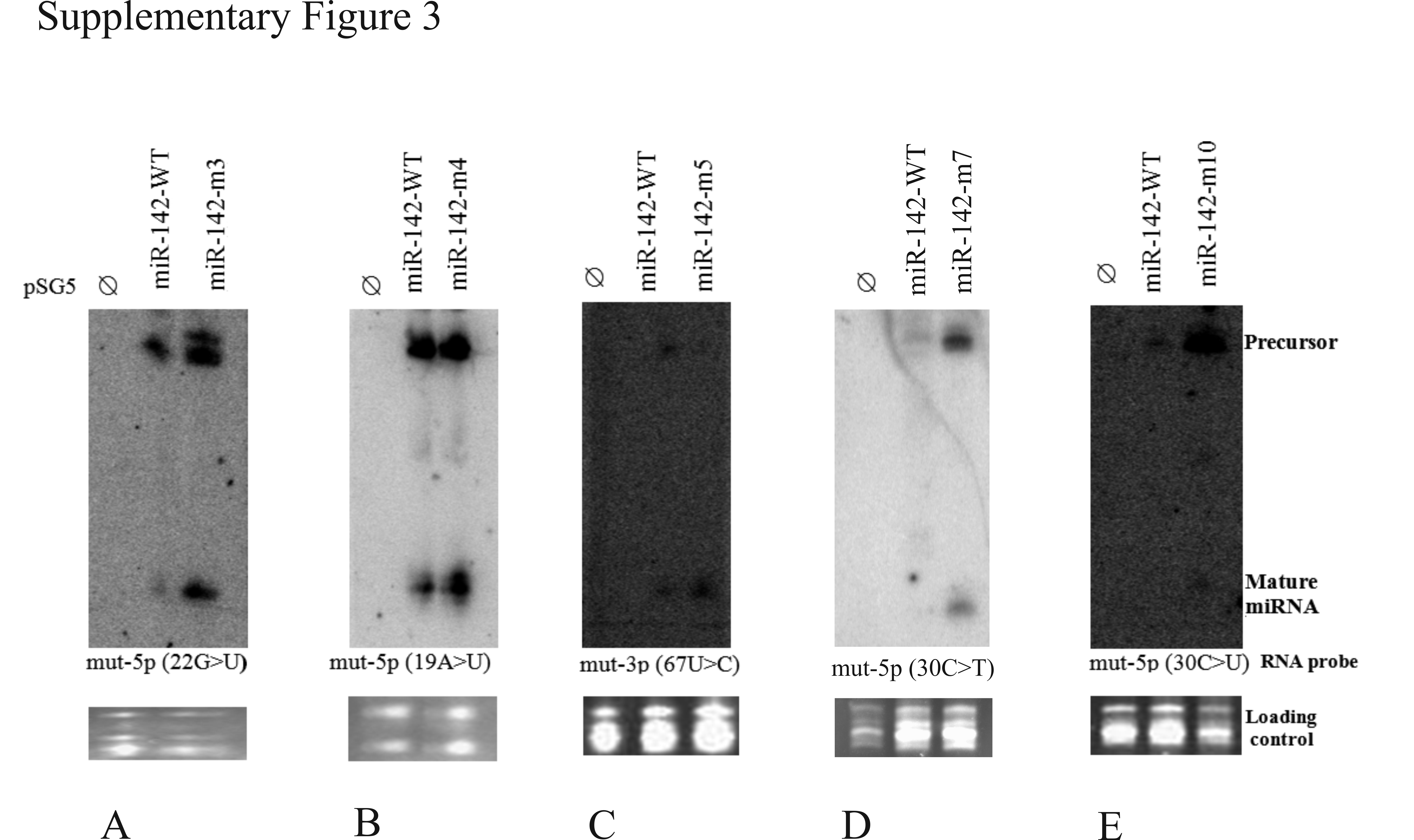

Supplement: Supplementary file 3 [file cam40001-0141-SD3.tif]

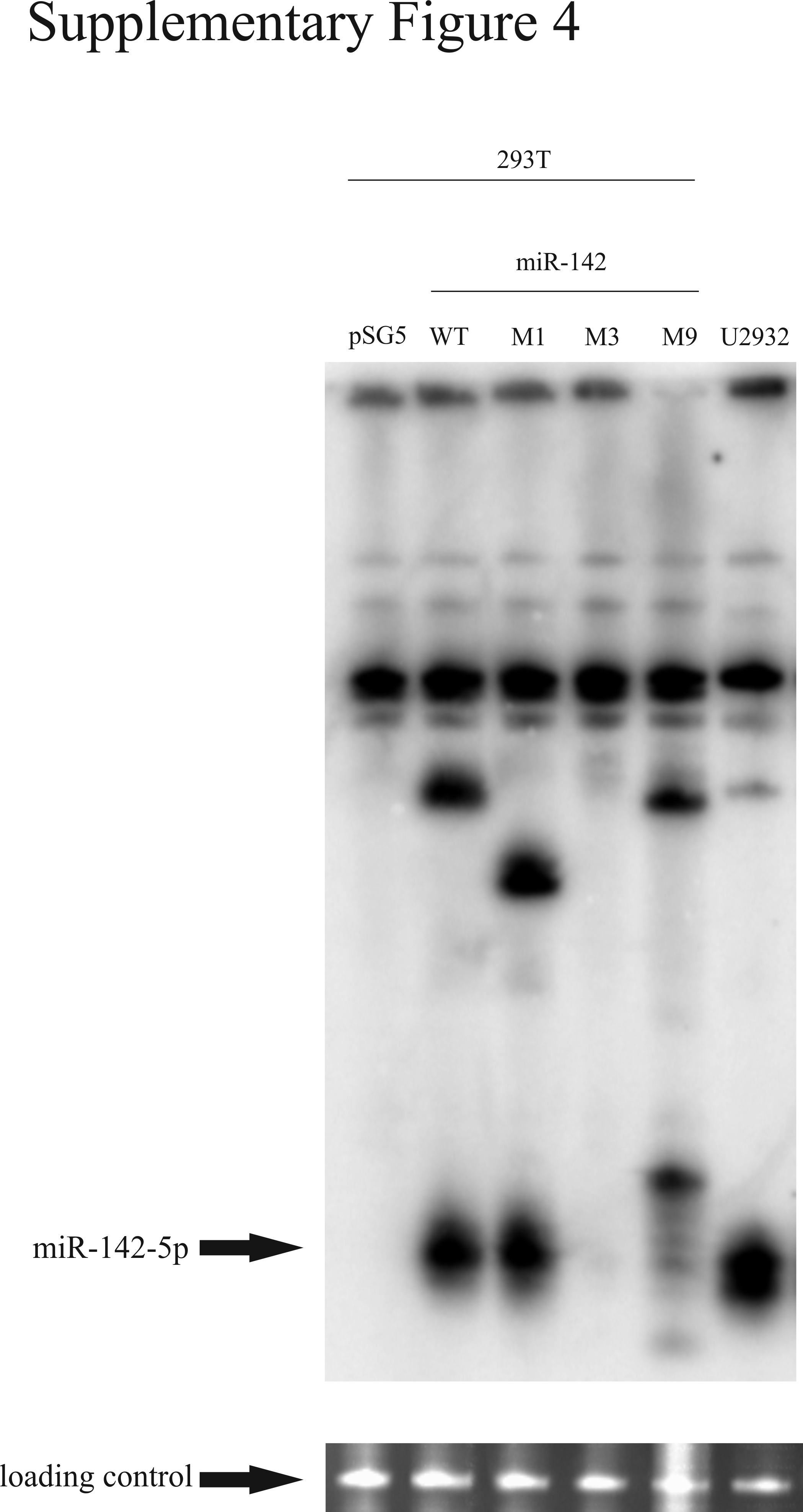

Supplement: Supplementary file 4 [file cam40001-0141-SD4.tif]

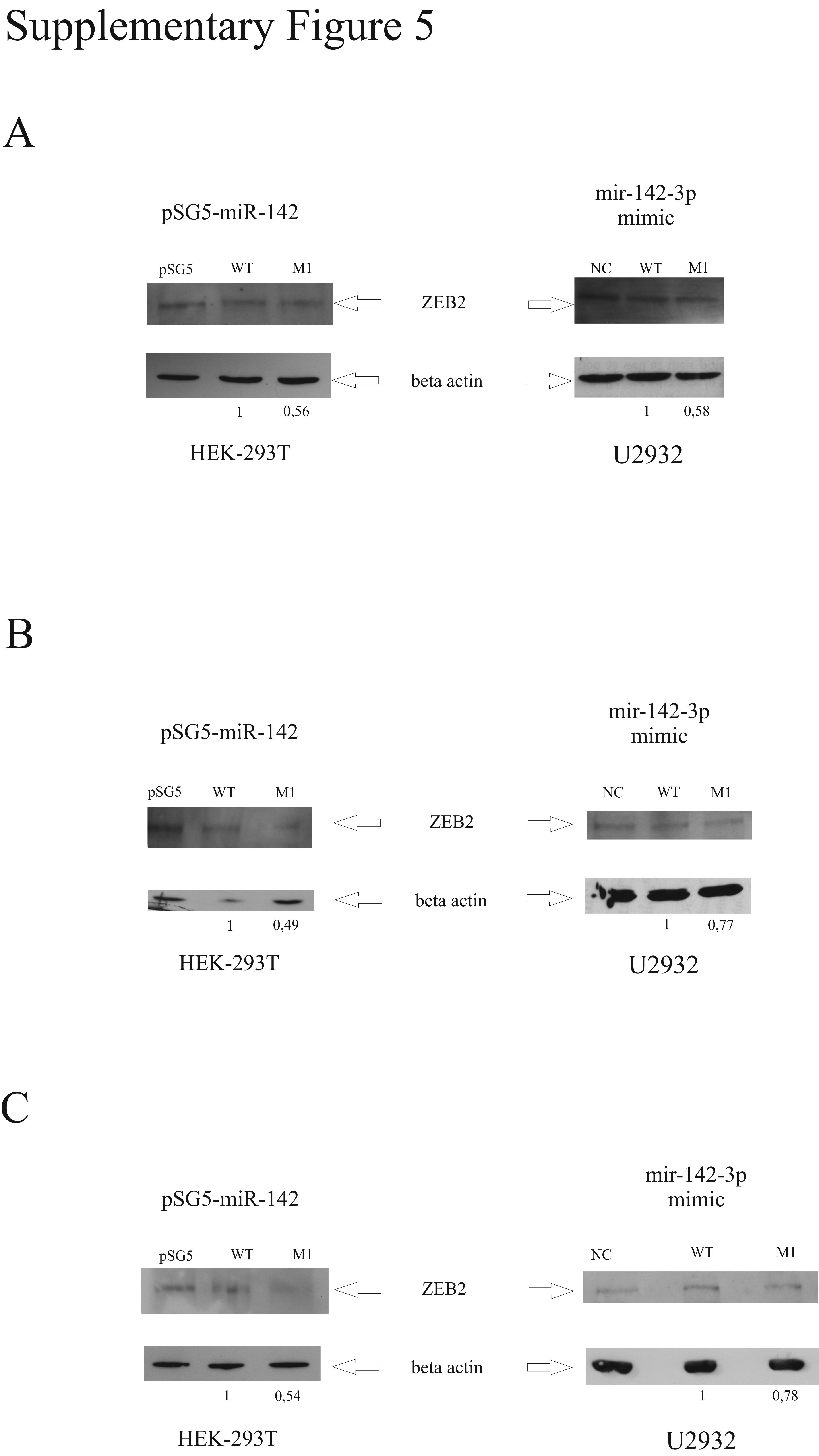

Supplement: Supplementary file 5 [file cam40001-0141-SD5.tif]

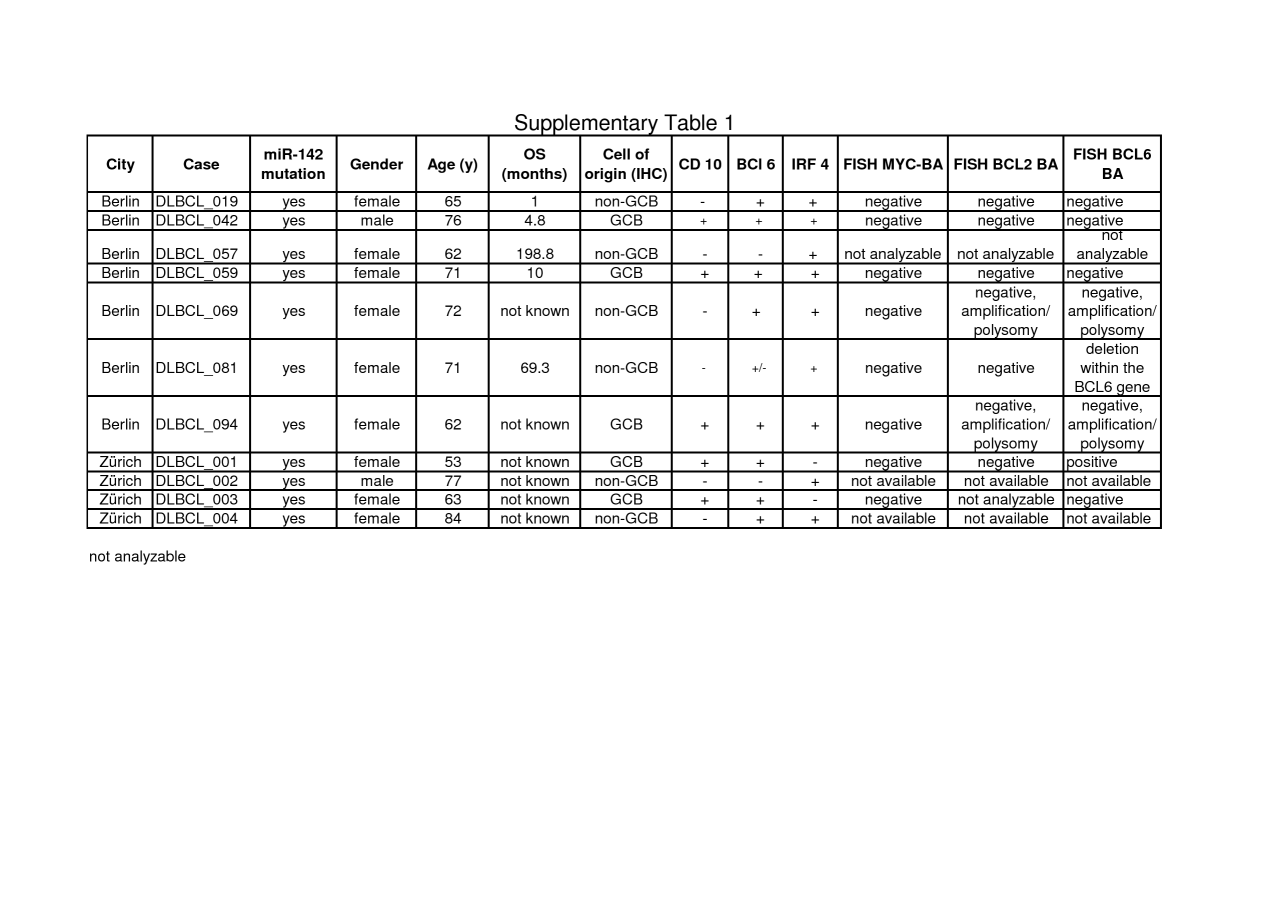

Supplement: Supplementary file 8 [file cam40001-0141-SD8.png]

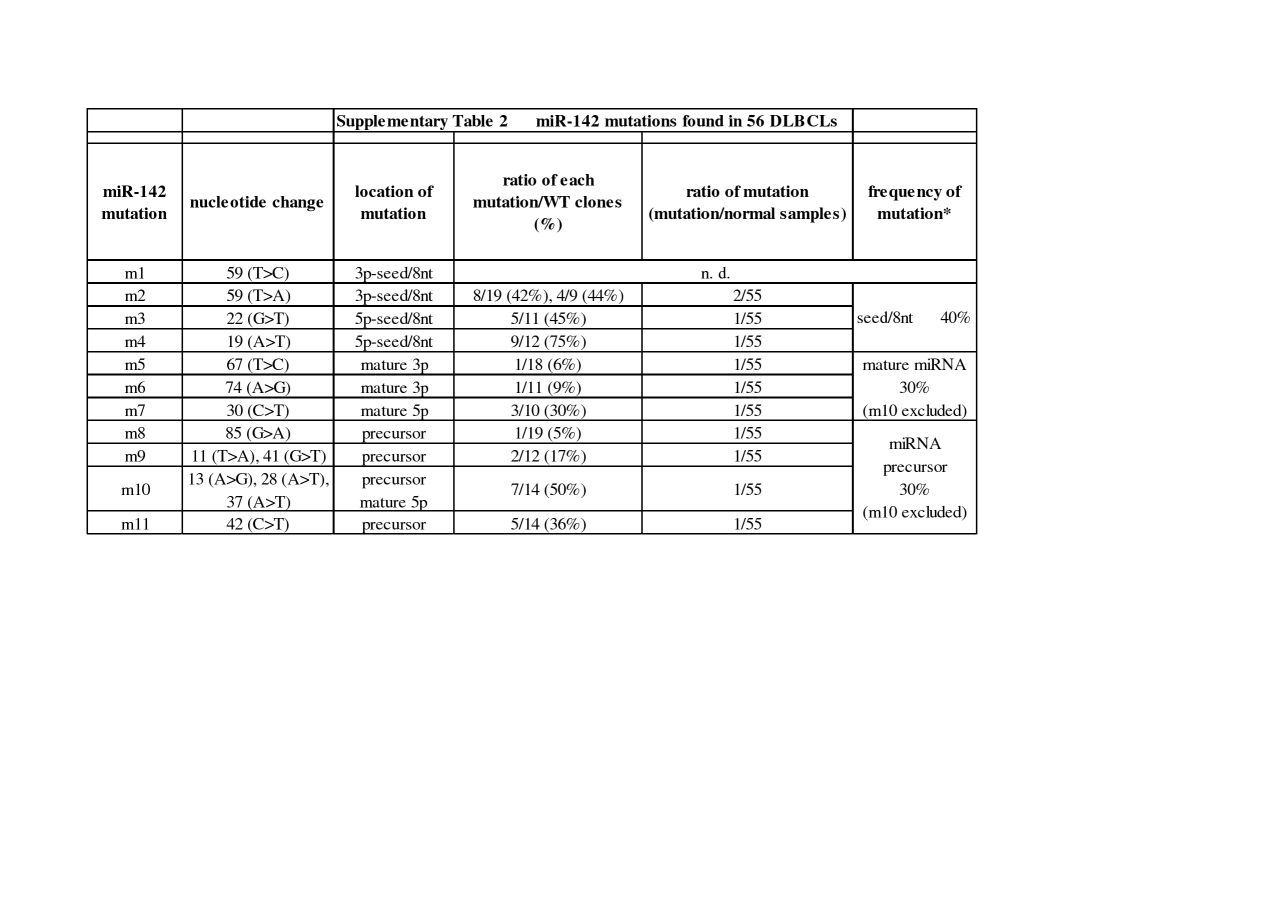

Supplement: Supplementary file 10 [file cam40001-0141-SD10.png]
