# Supplementary material for: MicroRNA-142 is mutated in about 20% of diffuse large B-cell lymphoma
Source: Cancer Med. 2012 Sep 18;1(2):141–55. doi: 10.1002/cam4.29 (PMC3544448; doi:10.1002/cam4.29)
Supplement: Supplementary file 7 [file cam40001-0141-SD7.pdf]

Supplementary Table 1

City	Case	miR-142 mutation	Gender	Age (y)	OS (months)	Cell of origin (IHC)	CD 10	Bcl 6	IRF 4	FISH MYC-BA	FISH BCL2 BA	FISH BCL6 BA
Berlin	DLBCL_019	yes	female	65	1	non-GCB	-	+	+	negative	negative	negative
Berlin	DLBCL_042	yes	male	76	4.8	GCB	+	+	+	negative	negative	negative
Berlin	DLBCL_057	yes	female	62	198.8	non-GCB	-	-	+	not analyzable	not analyzable	not analyzable
Berlin	DLBCL_059	yes	female	71	10	GCB	+	+	+	negative	negative	negative
Berlin	DLBCL_069	yes	female	72	not known	non-GCB	-	+	+	negative	negative, amplification/ polysomy	negative, amplification/ polysomy
Berlin	DLBCL_081	yes	female	71	69.3	non-GCB	-	+/-	+	negative	negative	deletion within the BCL6 gene
Berlin	DLBCL_094	yes	female	62	not known	GCB	+	+	+	negative	negative, amplification/ polysomy	negative, amplification/ polysomy
Zürich	DLBCL_001	yes	female	53	not known	GCB	+	+	-	negative	negative	positive
Zürich	DLBCL_002	yes	male	77	not known	non-GCB	-	-	+	not available	not available	not available
Zürich	DLBCL_003	yes	female	63	not known	GCB	+	+	-	negative	not analyzable	negative
Zürich	DLBCL_004	yes	female	84	not known	non-GCB	-	+	+	not available	not available	not available

not analyzable
