# Supplementary material for: MicroRNA-142 is mutated in about 20% of diffuse large B-cell lymphoma
Source: Cancer Med. 2012 Sep 18;1(2):141–55. doi: 10.1002/cam4.29 (PMC3544448; doi:10.1002/cam4.29)
Supplement: Supplementary file 9 [file cam40001-0141-SD9.pdf]

Supplementary Table 2 miR-142 mutations found in 56 DLBCLs

miR-142 mutation	nucleotide change	location of mutation	ratio of each mutation/WT clones (%)	ratio of mutation (mutation/normal samples)	frequency of mutation*
m1	59 (T>C)	3p-seed/8nt	n. d.		
m2	59 (T>A)	3p-seed/8nt	8/19 (42%), 4/9 (44%)	2/55	seed/8nt 40%
m3	22 (G>T)	5p-seed/8nt	5/11 (45%)	1/55	
m4	19 (A>T)	5p-seed/8nt	9/12 (75%)	1/55	
m5	67 (T>C)	mature 3p	1/18 (6%)	1/55	mature miRNA 30% (m10 excluded)
m6	74 (A>G)	mature 3p	1/11 (9%)	1/55	
m7	30 (C>T)	mature 5p	3/10 (30%)	1/55	
m8	85 (G>A)	precursor	1/19 (5%)	1/55	miRNA precursor 30% (m10 excluded)
m9	11 (T>A), 41 (G>T)	precursor	2/12 (17%)	1/55	
m10	13 (A>G), 28 (A>T), 37 (A>T)	precursor mature 5p	7/14 (50%)	1/55	
m11	42 (C>T)	precursor	5/14 (36%)	1/55	
